# Tracking reactive astrogliosis in autosomal dominant and sporadic Alzheimer’s disease with multi-modal PET and plasma GFAP

**DOI:** 10.1186/s13024-023-00647-y

**Published:** 2023-09-12

**Authors:** Konstantinos Chiotis, Charlotte Johansson, Elena Rodriguez-Vieitez, Nicholas J. Ashton, Kaj Blennow, Henrik Zetterberg, Caroline Graff, Agneta Nordberg

**Affiliations:** 1 Department of Neurobiology, Care Sciences and Society, Center for Alzheimer Research, Division of Clinical Geriatrics, Karolinska Institutet, Stockholm, Sweden; 2 Department of Neurology, Karolinska University Hospital, Stockholm, Sweden; 3 Department of Neurobiology, Care Sciences and Society, Center for Alzheimer Research, Division of Neurogeriatrics, Karolinska Institutet, Stockholm, Sweden; 4 Theme Inflammation and Aging, Karolinska University Hospital, Stockholm, Sweden; 5 Department of Psychiatry and Neurochemistry, Institute of Neuroscience and Physiology, The Sahlgrenska Academy, University of Gothenburg, Gothenburg, Sweden; 6 Centre for Age-Related Medicine, Stavanger University Hospital, Stavanger, Norway; 7 Institute of Psychiatry, Psychology & Neuroscience, King’s College London, Maurice Wohl Clinical Neuroscience Institute, London, UK; 8 NIHR Biomedical Research Centre for Mental Health & Biomedical Research Unit for Dementia at South London & Maudsley NHS Foundation, London, UK; 9 Clinical Neurochemistry Laboratory, Sahlgrenska University Hospital, Mölndal, Sweden; 10 Department of Neurodegenerative Disease, UCL Queen Square Institute of Neurology, University College London, London, UK; 11 UK Dementia Research Institute at UCL, London, UK; 12 Hong Kong Center for Neurodegenerative Diseases, Clear Water Bay, Hong Kong, China; 13 Wisconsin Alzheimer’s Disease Research Center, School of Medicine and Public Health, University of Wisconsin, University of Wisconsin-Madison, Madison, WI USA; 14 Unit for Hereditary Dementia, Karolinska University Hospital-Solna, Solna, Sweden

**Keywords:** Astrogliosis, Deprenyl, Plasma GFAP, Alzheimer’s disease, Astrocytes, Autosomal Dominant Alzheimer’s disease

## Abstract

**Background:**

Plasma assays for the detection of Alzheimer’s disease neuropathological changes are receiving ever increasing interest. The concentration of plasma glial fibrillary acidic protein (GFAP) has been suggested as a potential marker of astrocytes or recently, amyloid-β burden, although this hypothesis remains unproven. We compared plasma GFAP levels with the astrocyte tracer ^11^C-Deuterium-L-Deprenyl (^11^C-DED) in a multi-modal PET design in participants with sporadic and Autosomal Dominant Alzheimer’s disease.

**Methods:**

Twenty-four individuals from families with known Autosomal Dominant Alzheimer’s Disease mutations (mutation carriers = 10; non-carriers = 14) and fifteen patients with sporadic Alzheimer’s disease were included. The individuals underwent PET imaging with ^11^C-DED, ^11^C-PIB and ^18^F-FDG, as markers of reactive astrogliosis, amyloid-β deposition, and glucose metabolism, respectively, and plasma sampling for measuring GFAP concentrations. Twenty-one participants from the Autosomal Dominant Alzheimer’s Disease group underwent follow-up plasma sampling and ten of these participants underwent follow-up PET imaging.

**Results:**

In mutation carriers, plasma GFAP levels and ^11^C-PIB binding increased, while ^11^C-DED binding and ^18^F-FDG uptake significantly decreased across the estimated years to symptom onset. Cross-sectionally, plasma GFAP demonstrated a negative correlation with ^11^C-DED binding in both mutation carriers and patients with sporadic disease. Plasma GFAP indicated cross-sectionally a significant positive correlation with ^11^C-PIB binding and a significant negative correlation with ^18^F-FDG in the whole sample. The longitudinal levels of ^11^C-DED binding showed a significant negative correlation with longitudinal plasma GFAP concentrations over the follow-up interval.

**Conclusions:**

Plasma GFAP concentration and astrocyte ^11^C-DED brain binding levels followed divergent trajectories and may reflect different underlying processes. The strong negative association between plasma GFAP and ^11^C-DED binding in Autosomal Dominant and sporadic Alzheimer’s disease brains may indicate that if both are markers of reactive astrogliosis, they may detect different states or subtypes of astrogliosis. Increased ^11^C-DED brain binding seems to be an earlier phenomenon in Alzheimer’s disease progression than increased plasma GFAP concentration.

**Supplementary Information:**

The online version contains supplementary material available at 10.1186/s13024-023-00647-y.

## Introduction

There is a growing body of literature, which recognises that astrocyte reactivity (reactive astrogliosis) is a central feature in the pathophysiology of Alzheimer’s disease and other neurodegenerative diseases [1–3]. Astrocytes become reactive in response to changes in brain homeostasis and they adopt heterogeneous activation states by cell remodelling in a dynamic manner [4, 5]. However, the role of the different states in the neurodegeneration cascade appears complex [4]. While some states have a homeostatic function, others might have a detrimental role. The functional and morphological characteristics of the reactive states, as well as the respective gene expression profiles [5], allow for development of a diverse palette of biomarkers that bring potential to increase understanding of the functional heterogeneity of astrocyte reactivity.

Positron emission tomography tracers targeting the enzyme monoamine oxidase B (MAO-B) [6, 7] or the closely associated imidazoline I2 receptor [8], have allowed the in vivo imaging of a subtype of reactive astrocytes which overexpress the MAO-B enzyme. We have previously reported higher binding of the MAO-B tracer ^11^C-Deuterium-L-Deprenyl (DED) in patients with sporadic Alzheimer’s disease at the mild cognitive impairment (MCI) stage of the disease [9], relative to healthy individuals. Furthermore, our observations from multi-tracer PET studies in autosomal dominant Alzheimer’s disease (ADAD) indicated that the ^11^C-DED binding declines from a level of increased binding in presymptomatic mutation carriers starting at about 17 years before the estimated time of symptom onset [7, 10, 11]. Together, evidence from sporadic Alzheimer’s disease and ADAD stresses that changes in reactive astrogliosis happen early in the disease process [12]. These data are consistent with our findings in the transgenic APPswe mouse models of Alzheimer’s disease where high ^11^C-DED binding was observed in 6 months old mice and lower binding in 18–24 months old mice [13]. In contrast the levels of amyloid-beta plaques and glial fibrillary acidic protein (GFAP) exhibited the opposite pattern and were found to be high in 18–24 month old mice.

GFAP immunostaining is the most widely used marker for detecting in vitro reactive astrocytes. However, although GFAP, which is a cytoskeletal protein, is thought to be released by injured astrocytes, it is not a universal reactivity marker [5, 14]. Assays targeting plasma GFAP have recently emerged and tested in large imaging cohorts [15]. The available evidence supports that high plasma GFAP concentration could be associated with the presence of amyloid-β pathology and that plasma GFAP levels are high in the initial stages of sporadic Alzheimer’s disease [16–18]. In ADAD, increases in plasma GFAP levels are reported at the presymptomatic stage of the disease [19–21]. However, there is a paucity of evidence assessing the relationship between plasma GFAP concentration and the activation of brain astrocytes.

The aim of this study was to evaluate the association between plasma GFAP levels and brain reactive astrogliosis as measured with ^11^C-DED in a multi-modal PET design in presymptomatic and symptomatic ADAD mutation carriers and patients with sporadic Alzheimer’s disease.

## Materials and methods

### Study design and participants

Participants from families with known ADAD mutations and sporadic patients with cognitive impairment were included in this study, as detailed below. The participants in the present study form a subset of individuals from our previously published studies. For a more comprehensive understanding of the study design, additional information can be found in our earlier publications [9, 10, 19].

The sporadic patients with cognitive impairment were referred for cognitive assessment to the Memory clinic, Theme Inflammation and Aging, Karolinska University Hospital Huddinge, Stockholm, Sweden. All underwent an extensive clinical assessment including neuropsychological testing, MRI imaging and CSF sampling [10]. As part of a research protocol, the patients also underwent multi-modal PET investigations. Eleven patients qualified for a clinical diagnosis of mild cognitive impairment and had evidence of amyloid-β pathology (MCI+, prodromal Alzheimer’s disease) [22, 23], four received a diagnosis of Alzheimer’s disease dementia with evidence of amyloid-β pathology [24] and four additional patients received a diagnosis of MCI without evidence of amyloid-β pathology (MCI-). For a more comprehensive understanding of the group classification, we refer readers to the Supplementary Information section and to the work of Rodriguez-Vieitez et al. [10]. For the group analyses, the fifteen patients with cognitive impairment and evidence of amyloid-β pathology (i.e. MCI + and Alzheimer’s disease dementia) were allocated together in a sporadic Alzheimer’s disease group (n = 15) [23].

The ADAD participants in this study are part of a long-term ongoing longitudinal observational research study at Karolinska Institutet and include families carrying one of four mutation types, each with a mutation-specific average age of expected symptom onset. Twenty-three individuals from families with known ADAD mutations in the *PSEN1* (p.H163Y and p.I143T) and *APP* (p.KM670/671NL, *APP*swe and p.E693G, *APP*arc) genes (9 mutation carriers, 14 non-carriers) volunteered and underwent an extensive clinical and neuropsychological evaluation, MRI, plasma and CSF sampling and multi-modal PET imaging [10]. All clinicians and researchers which were in contact with the ADAD-family members were blind to the mutation status. Mutation carriers were either symptomatic (sMC, CDR-SOB > 0) or presymptomatic (pMC, CDR-SOB < 0). Non-carriers from the different families were included as a common reference group. For a given mutation, the age of expected symptom onset was defined as the average age at which individuals from a specific family developed the first clinically relevant cognitive symptoms. One presymptomatic mutation carrier was excluded from all analyses because of no penetrance of the mutation; the individual passed the estimated age of symptom onset by more than a decade, showing no symptoms or evidence of amyloid-β pathology in PET [25].

### Imaging acquisition and quantification

PET imaging acquisitions were performed with ^11^C-DED for reactive astrogliosis, ^11^C-PIB for amyloid-β, and ^18^F-FDG for glucose metabolism, as previously detailed [10]. The participants underwent extensive MRI acquisitions.

The T1 MRI images were segmented in SPM12 and individual grey matter masks were created. An in-house volumetric Montreal Neurological Institute space version of the Desikan-Killiany atlas [26] was warped to the participant’s MRI space based on the inverse deformation field from the segmentation step. The individual grey matter masks were used for identifying grey matter regions of interest (ROI) in the atlas in the native MRI space. Finally, the dynamic PET images were co-registered to the T1 MRI images with SPM12 for performing the tracer quantification.

Briefly, dynamic acquisitions (0–60 min) were performed for all tracers. ^11^C-PIB binding and ^18^F-FDG uptake was quantified with the use of standard uptake value ratio (SUVR) images (40–60 and 30–45 min, respectively), relative to the binding/uptake in the pons. ^11^C-DED binding was quantified as the slope from the Patlak reference tissue model, as applied in the open-source QModeling toolbox [27], after modifying the time-activity curve of the reference region (cerebellar grey matter including the dentate nucleus area) in order to obtain linear slopes for the Patlak plot. This approach was selected to account for MAO-B in the reference region and is detailed elsewhere [28]. To assess the validity of static quantification (SUVR) for ^11^C-DED binding, we evaluated its comparability with the slope binding measures obtained from the Patlak reference tissue model. Detailed data regarding this comparison are presented in the Supplementary Information section. Based on previous studies, the evidence for ^11^C-DED, ^11^C-PIB and ^18^F-FDG [10] was summarized in composite temporal, global cortical (Centiloid ROI [29]) and temporoparietal ROIs, respectively. The resulting ^11^C-PIB SUVR for the Centiloid global cortical ROI was converted to Centiloids using standard procedures [29].

### Plasma sampling and quantification of GFAP levels

Non-fasting whole blood samples were collected by venipuncture, using sodium heparin additives. Samples were centrifuged for 10 min at 2200 g at room temperature, within an hour of sampling, and the supernatant plasma was aliquoted into 1ml polypropylene tubes and frozen at -80^0^C [19].

Plasma GFAP concentration was measured using an ultra-sensitive immunoassay technology, the Quanterix Simoa™ Human Neurology 4-plex A Assay (Quanterix Corporation, Billerica, MA. The lower limit of quantification is 0.467pg/mL, and a pooled coefficient of variation for the assay is 12.9%. At the time points where both PET investigations and plasma sampling were available, the median interval between the two was 3 months (interquartile range = 2–5 months). The samples from the ADAD and sporadic participants were analysed at two different time points using the same quality control to account for potential batch-to-batch variation.

Although studies suggest the GFAP assays are robust and are resistant to handling variations [30, 31], the test-retest stability and longitudinal fluctuation in plasma GFAP concentration at the individual level remains unclear. This is of particular importance in this study, given that potential single outlier plasma GFAP values – at an intra-individual level – at the time points when concurrent PET data is available could have a considerable effect on the evaluation of the association between markers. To that end, intraindividual variability analysis was conducted in three steps. Firstly, the longitudinal plasma GFAP concentration was qualitatively assessed for all individuals with more than two time points to identify values that deviated from their expected longitudinal trajectory. Secondly, slopes were calculated for each individual’s trajectory, and these resulting slopes were compared to detect any deviating trajectories. Lastly, the residual values from the linear mixed-effects model, used to model the longitudinal trajectory of plasma GFAP concentrations for all individuals, were screened following standard procedures to identify potential values with unexplained variability by the model. As a result of this analysis, one baseline plasma GFAP value in the dataset (GFAP = 257 pg/mL at baseline) was found to be inconsistent with the individual’s longitudinal trajectory (GFAP = 113 pg/mL four years from baseline, and 132 pg/mL six years from baseline) and was subsequently excluded from the analysis as it appeared implausible. Further details can be found in the Supplementary Information section.

### Statistical analyses

Linear mixed-effects models were applied for modelling the longitudinal trajectories of the different biomarkers (^11^C-DED, ^11^C-PIB and ^18^F-FDG, and plasma GFAP concentrations) in separate models in mutation carriers and non-carriers from the ADAD participants, with estimated years from symptom onset as a fixed-effect and a random intercept that accounted for repeated measures at the individual level. For the analyses pertaining to ^11^C-PIB binding, *APP*arc mutation carriers were excluded due to the known mutation-specific relative sparsity of fibrillar amyloid-β that causes exceptionally low ^11^C-PIB binding levels [32]. P values were quantified with the Satterthwaite’s degrees-of-freedom method.

To address the known non-linear trajectories of the individual biomarkers in the ADAD mutation carriers [33], we performed a second exploratory analysis applying generalized additive mixed models. This is a technique where the impact of the predictive variables is captured through smooth functions and can therefore handle highly non-linear relationships between the response and explanatory variables, with no a priori assumptions for the exact shape of the non-linear relationship. Fixed and random effects were set identically for the generalized additive mixed and linear mixed-effects models. For allowing comparisons between levels of different markers in terms of trajectories, we centred the binding/uptake and plasma GFAP values of the mutation carriers based on the mean value of the non-carriers for each biomarker, and then transformed the values based on the standard deviation for each biomarker (i.e., standardized difference from non-carriers), similarly to Bateman et al. (2012) [33].

The association between each PET tracers’ binding/uptake and plasma GFAP concentration was assessed (1) Cross-sectionally with the Spearman’s correlation coefficient and (2) Longitudinally with linear mixed-effects models. In the mixed-effects models, plasma GFAP concentration was set as the dependent variable, PET tracers’ binding/uptake as the fixed-effect, and a random intercept was used to account for repeated measures at the individual level. Separate analyses were performed for non-carriers, ADAD mutation carriers and patients with sporadic Alzheimer’s disease (only cross-sectional data were available). Spearman partial correlations were additionally employed to assess the cross-sectional association between ^11^C-DED PET tracer binding and plasma GFAP concentration, after adjustment for age and gender, in the groups of ADAD mutation carriers, non-carriers and patients with sporadic Alzheimer’s disease.

All the above statistical analyses were carried out using R 4.2.1 software. All tests were 2-tailed, with a significance level of α = 0.05.

## Results

### Study participants

The sample characteristics, with demographic data, the follow-up interval for each biomarker and the number of follow-up investigations are shown in Table 1.

**Table 1:** Sample characteristics

	Non-carriers	ADAD mutation carriers		Sporadic Alzheimer’s disease		MCI-
		Presymptomatic	Symptomatic	MCI+	AD	
**n at baseline**	14 ^1^	6 ^2^	4 ^3^	11	4	4
**Estimated years from symptom onset at baseline PET**	2 [-4 : 9]	-7 [-11 : -4]	1, 3, 6, 8	-	-	-
**Age at baseline PET**	56 [45 : 63]	44 [42 : 47]	55, 57, 58, 64	60 [57: 67]	56, 67, 67, 70	54, 60, 61, 72
**Plasma GFAP concentration at baseline PET**	138 [65 : 161]	116 [84 : 141]	71, 285 ^4^	118 [100 : 140]	117, 137, 196, 275	76, 78, 95, 144
**Follow-up interval for PET (years)**	3.1 [2.6 : 3.7]	2.5 [2.4 : 2.9]	2.8	-	-	-
**n with follow-up PET (number of total follow-up investigations)**	4 (4)	5 (5)	1 (1)	-	-	-
**Follow-up interval for plasma GFAP (years)**	14.8 [5.3 : 15.7]	16.3 [6.9 : 21.0]	15.5, 18.2	-	-	-
**n with follow-up plasma sampling (number of total follow-up investigations)**	13 (22)	6 (22)	2 (9)	-	-	-

The data are presented as median [interquartile range], unless otherwise indicated. In groups with n < 5, the individual values for each variable are presented

^1^ C-PIB data were not available for one participant (n = 1)

^2^ One participant was carrier of the APParc mutation (n = 1)

^3^ Two participants were carriers of the APParc mutation (n = 2)

^4^ Plasma GFAP measures were not available for two participants (n = 2)

NC: non-carriers; pMC: presymptomatic mutation carriers; sMC: symptomatic mutation carriers; MCI-: MCI with a negative amyloid-β PET scan; MCI+: MCI with a positive amyloid-β PET scan; AD: Alzheimer’s disease dementia

### Longitudinal trajectories of biomarkers in ADAD mutation carriers

**Fig. 1:**
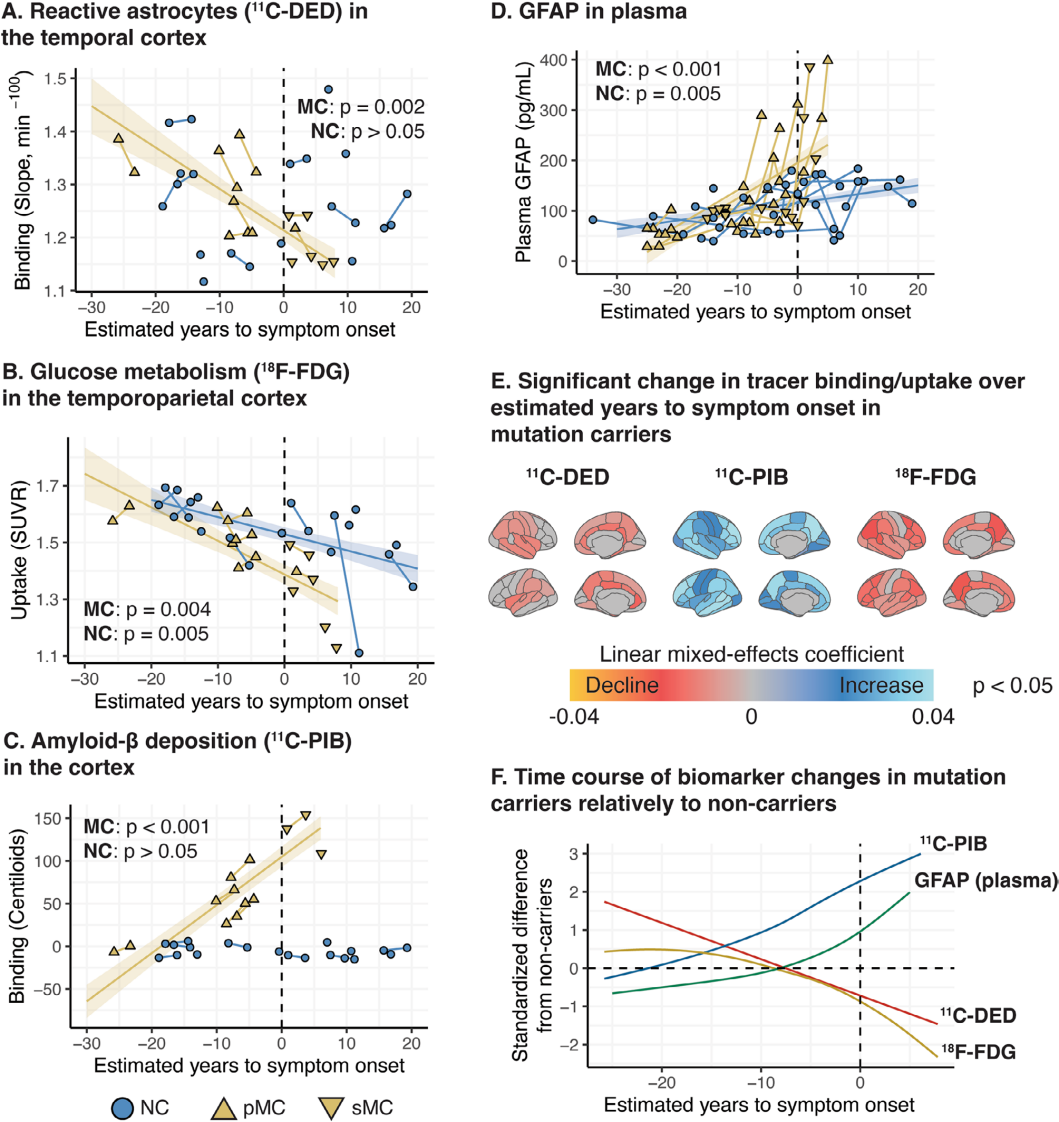
Longitudinal trajectories of biomarker changes based on mixed-effects modelling in ADAD mutation carriers. **(A-D)** Regression plots illustrating the association between estimated time to expected symptom onset and biomarker level in ADAD mutation carriers (linear mixed-effects models). **(E)** Surface maps depicting the ROIs with a significant change in the PET binding/uptake values over the estimated time to expected symptom onset in ADAD mutation carriers. **(F)** Regression plots comparing the longitudinal non-linear trajectories of the different biomarkers in ADAD mutation carriers across the estimated time to expected symptom onset (generalized additive mixed-effects models); the biomarker values are presented as standardised difference from non-carriers (NC). For the generalized additive mixed model **(F)** we illustrate the ^11^C-DED, ^11^C-PIB and ^18^F-FDG binding/uptake values in the composite temporal, global cortical and temporoparietal ROIs, respectively. The p value of the models is shown (A-D). pMC: presymptomatic mutation carriers; sMC: symptomatic mutation carriers. In Fig. 1A, B, D, an ADAD mutation carrier is denoted by a normal triangle, even though the individual surpassed the baseline point (0) on the estimated years to symptom onset axis. This discrepancy is due to the fact that the participant did not exhibit any signs of cognitive impairment at that time, and this inconsistency falls within the expected error between the estimated age of symptom onset and the actual age of symptom onset at the individual level. Subsequently, during the follow-up assessment, this same participant showed clear objective evidence of cognitive impairment, as indicated by the use of an inverted triangle. For the analyses pertaining to ^11^C-PIB binding, *APP*arc mutation carriers were excluded due to the known mutation-specific relative sparsity of fibrillar amyloid-β that causes exceptionally low ^11^C-PIB binding levels

^11^C-DED binding in the temporal ROI and ^18^F-FDG uptake in the temporoparietal ROI declined significantly across the estimated time to symptom onset in ADAD mutation carriers (Fig. 1A, B). Similarly, the ^11^C-PIB binding in the global cortical ROI and the plasma GFAP concentration increased significantly across the estimated time to symptom onset in ADAD mutation carriers (Fig. 1C, D). When assessing all individual cortical ROIs, the decline in ^11^C-DED binding (reactive astrogliosis) was predominantly detected in the temporoparietal and the cingulate cortical ROIs, and the decline in ^18^F-FDG (glucose metabolism) in parietal cortical ROIs (Fig. 1E). The increases in ^11^C-PIB binding (amyloid-β deposition) were detected in all frontotemporoparietal cortical association ROIs (Fig. 1E).

Based on the exploratory analysis results that allow for non-linear modelling and relatively to non-carriers, mutation carriers showed high ^11^C-DED and ^11^C-PIB binding at an earlier estimated time to symptom onset compared to when increase in plasma GFAP concentration and decline in ^18^F-FDG uptake were detected in the same individuals (Fig. 1F). More specifically, the GFAP plasma concentration started to increase above the mean non-carrier level later than when high ^11^C-DED was detectable and the GFAP plasma concentration peaked close to symptom onset (Fig. 1F).

### Baseline group comparisons

**Fig. 2:**
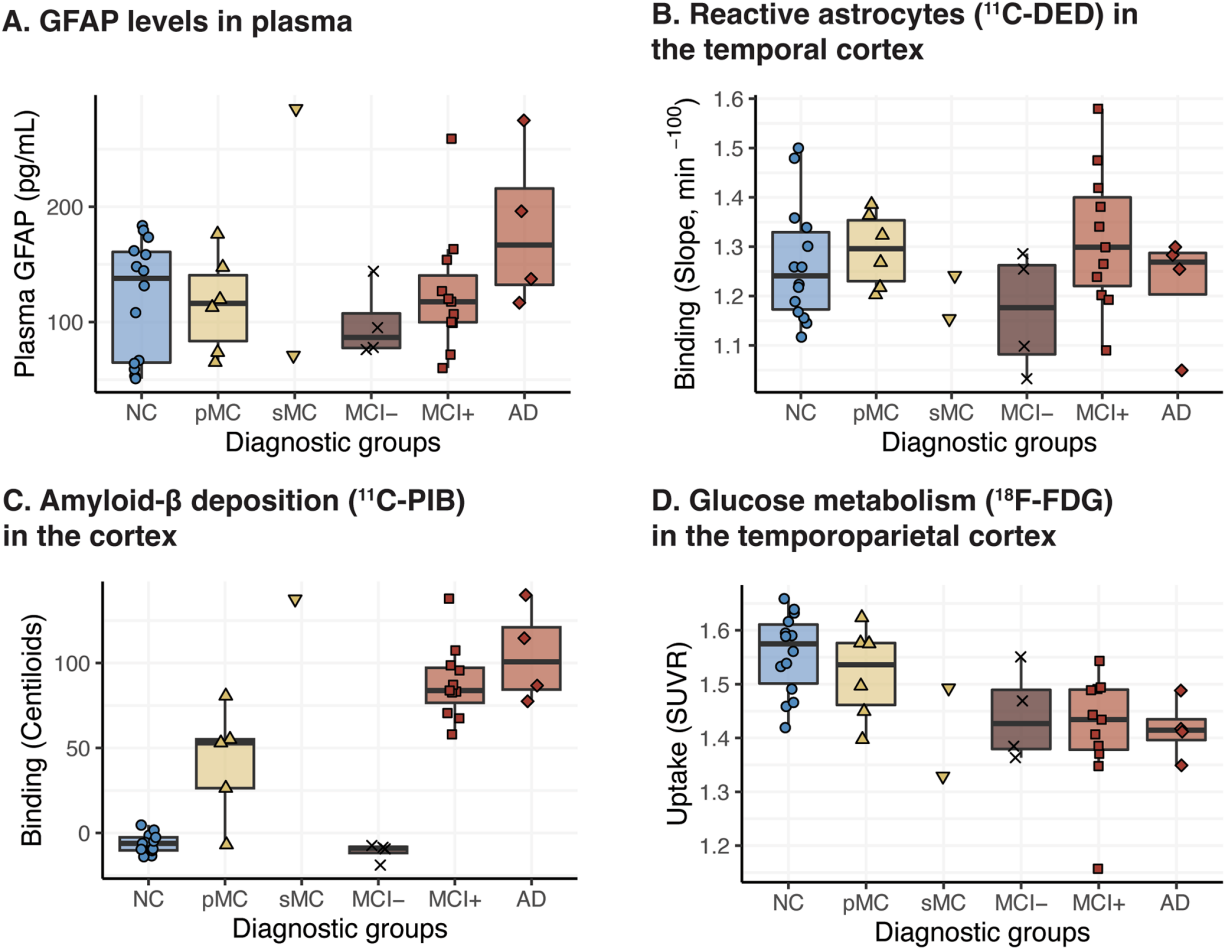
Boxplots of the biomarker levels in the different diagnostic groups. Inferential statistics were not used for group comparisons due to the small size of the individual groups. For the analyses pertaining to ^11^C-PIB binding, *APP*arc mutation carriers were excluded due to the known mutation-specific relative sparsity of fibrillar amyloid-β that causes exceptionally low ^11^C-PIB binding levels. NC: non-carriers; pMC: presymptomatic mutation carriers; sMC: symptomatic mutation carriers; MCI-: MCI with a negative amyloid-β PET scan; MCI+: MCI with a positive amyloid-β PET scan; AD: Alzheimer’s disease dementia

The extent of plasma GFAP and ^11^C-DED binding followed a different pattern across diagnostic groups at baseline. Thus, high plasma GFAP concentration was detected mainly in patients with Alzheimer’s disease dementia (Fig. 2A) while high ^11^C-DED binding was detected mainly in presymptomatic ADAD mutation carriers and patients with MCI+ (Fig. 2B). Elevated ^11^C-PIB binding corresponded with lower levels of ^18^F-FDG uptake, as shown in Fig. 2C, D across the different diagnostic groups. We did not use inferential statistics for group comparisons because of the small size of the individual groups.

### Cross-sectional associations between PET biomarkers and plasma GFAP

**Fig. 3:**
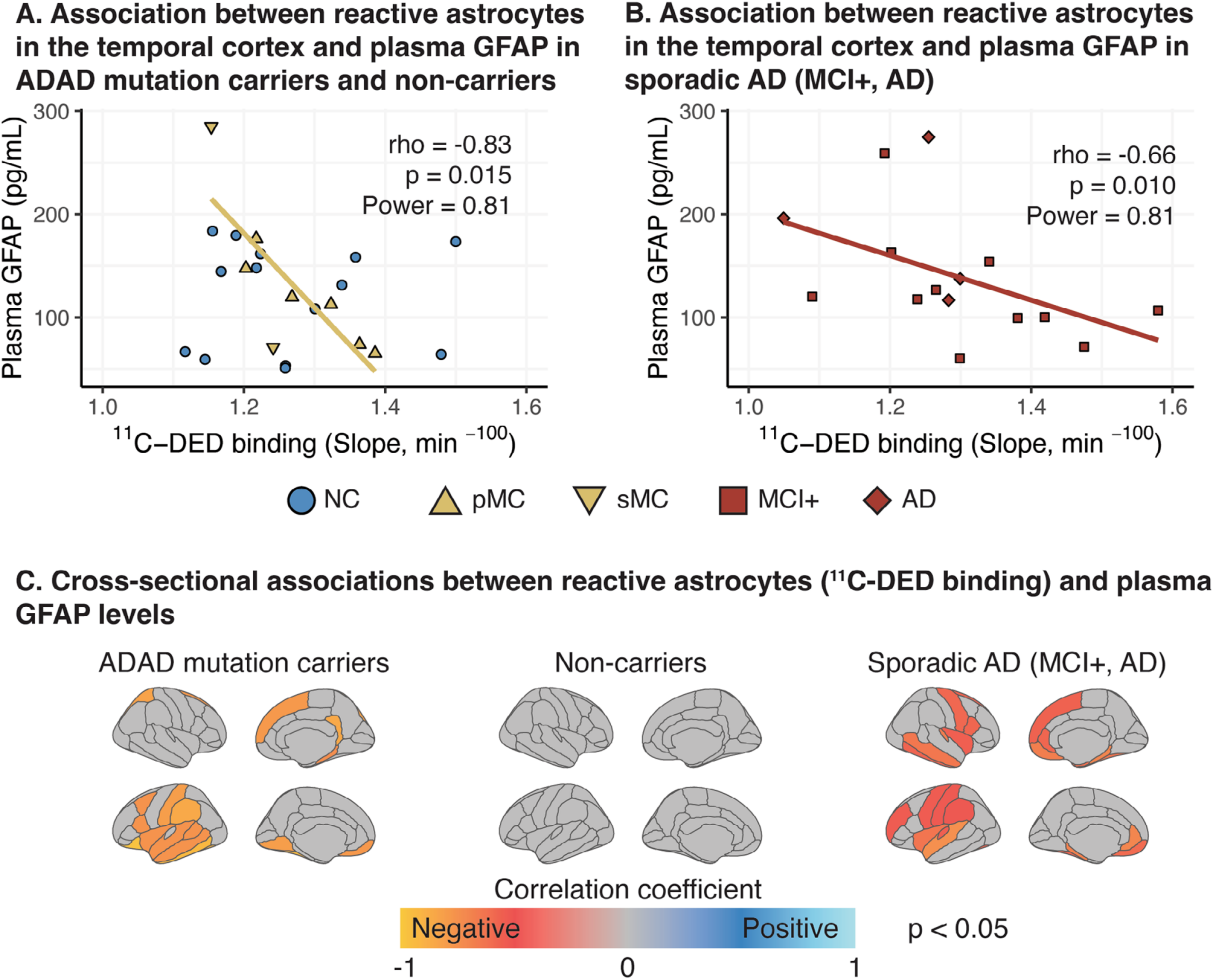
Cross-sectional correlation analyses between ^11^C-DED PET binding and plasma GFAP levels. **(A-B)** Scatterplots evaluating the relationship between ^11^C-DED PET binding and plasma GFAP levels in carriers of ADAD mutations and patients with sporadic Alzheimer’s disease for composite temporal ROIs, and **(C)** surface maps illustrating in colour the cortical ROIs with a significant cross-sectional association between ^11^C-DED PET binding and plasma GFAP. The Spearman’s correlation coefficient (rho), the p and power values are shown. NC: non-carriers; pMC: presymptomatic mutation carriers; sMC: symptomatic mutation carriers; MCI+: MCI with a positive amyloid-β PET scan; AD: Alzheimer’s disease dementia

^11^C-DED binding in the temporal cortex and plasma GFAP concentration showed significant strong negative correlations in both ADAD mutation carriers and patients with sporadic Alzheimer’s disease (Fig. 3A, B). When assessing ^11^C-DED binding in all individual cortical ROIs, significant negative correlations with plasma GFAP concentration were detected in widespread, mainly frontotemporal cortical ROIs, in both ADAD mutation carriers and patients with sporadic Alzheimer’s disease separately (Fig. 3C). In non-carriers, no correlation was observed between ^11^C-DED binding and plasma GFAP (Fig. 3A, C). Similar results were obtained from the partial correlation analysis after adjustment for age and gender, although the areas with significant correlations were restricted to fewer ROIs in the ADAD mutation carrier group (Supplementary Information).

**Fig. 4:**
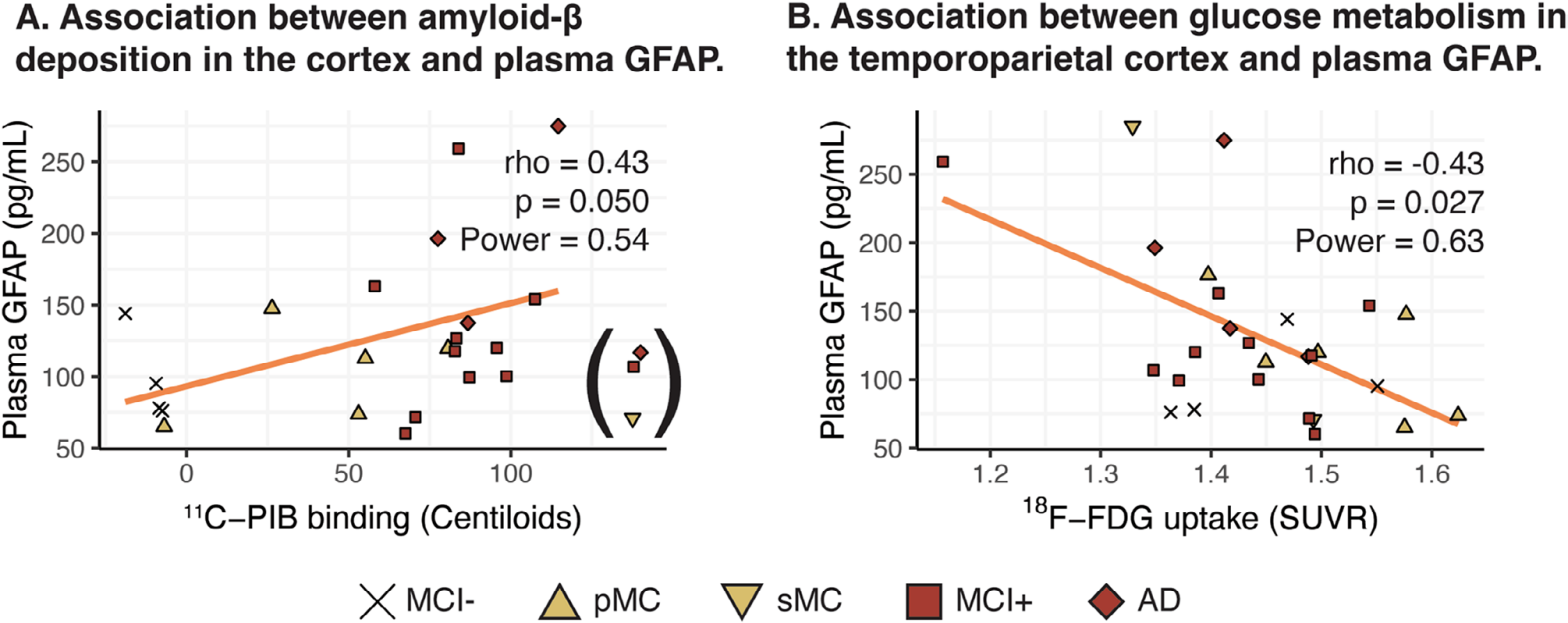
Cross-sectional correlation analyses evaluating the relationship between ^11^C-PIB, ^18^F-FDG PET tracers’ binding/uptake and plasma GFAP. The analyses were performed for composite cortical ROIs **(A, B)** in the whole sample, not considering the non-carrier group. For the correlation analyses between ^11^C-PIB binding and plasma GFAP levels, the individuals with extremely high ^11^C-PIB binding (marked in parentheses in the figures) were excluded given the known non-linear association between the biomarkers in high amyloid-β levels [48]. For the analyses pertaining to ^11^C-PIB binding, *APP*arc mutation carriers were excluded due to the known mutation-specific relative sparsity of fibrillar amyloid-β that causes exceptionally low ^11^C-PIB binding levels. The Spearman’s correlation coefficient (rho), the p and power values are shown. NC: non-carriers; pMC: presymptomatic mutation carriers; sMC: symptomatic mutation carriers; MCI-: MCI with a negative amyloid-β PET scan; MCI+: MCI with a positive amyloid-β PET scan; AD: Alzheimer’s disease dementia

^11^C-PIB binding in the global cortical ROI and plasma GFAP concentration showed a significant positive correlation in the whole sample, when excluding the non-carriers group (high variability in plasma GFAP concentration with a negative ^11^C-PIB scan) as well as some few individuals (n = 3) with very high ^11^C-PIB binding levels and low corresponding plasma GFAP concentration (Fig. 4A). ^18^F-FDG uptake in the temporoparietal ROI and plasma GFAP concentration showed a significant negative correlation in the whole sample, when excluding the non-carriers group (Fig. 4B).

### Longitudinal associations between PET biomarkers and plasma GFAP in ADAD participants

**Fig. 5:**
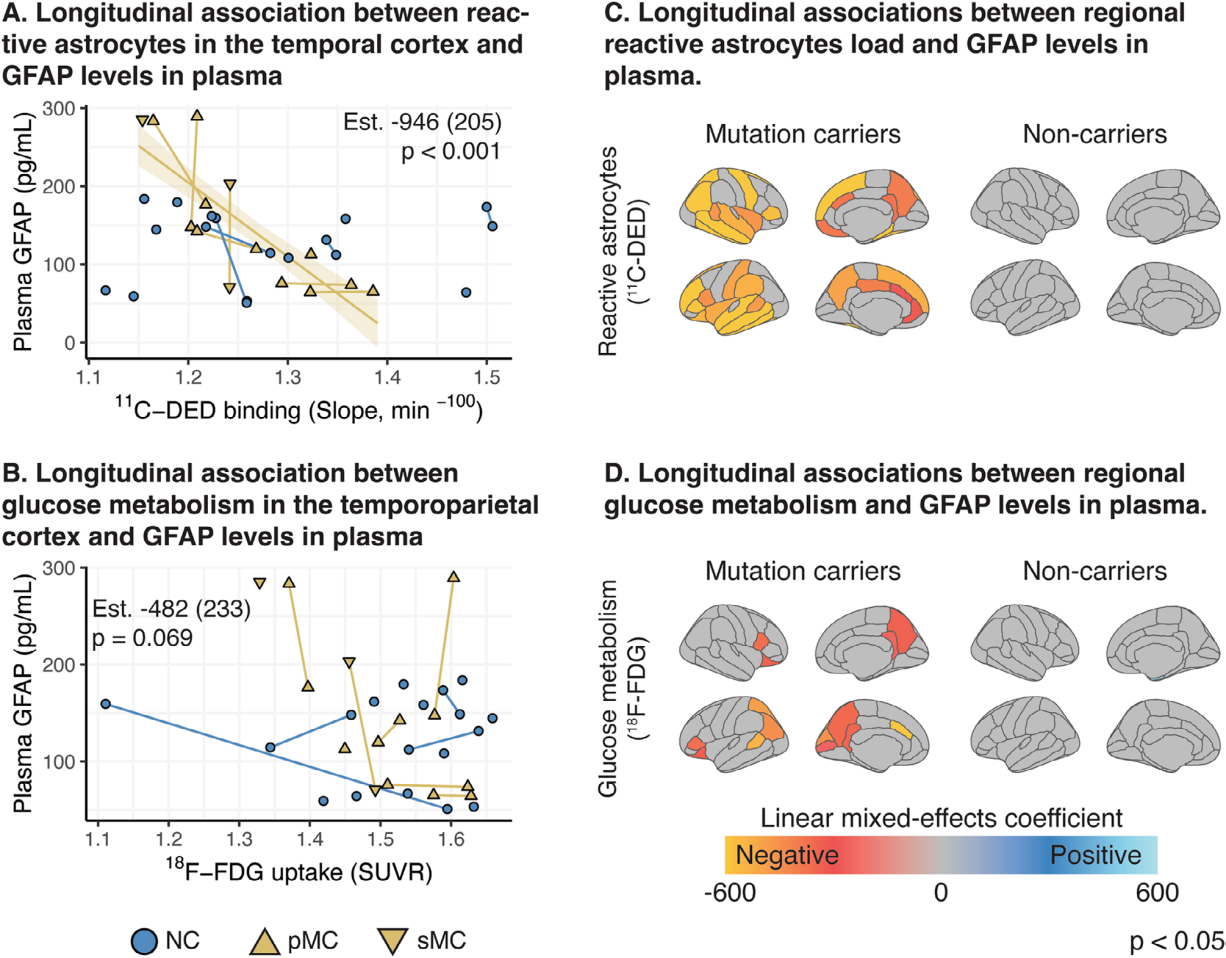
Longitudinal linear mixed-effects model analyses evaluating the relationship between PET tracers’ binding/uptake and plasma GFAP. **(A, B)** Scatterplots for composite cortical ROIs and **(C, D)** surface maps where cortical ROIs with a significant longitudinal association between PET tracers’ binding/uptake and plasma GFAP are depicted in colour. The estimate (Est.), the standard error (parentheses) and p value of the models are shown. NC: non-carriers; pMC: presymptomatic mutation carriers; sMC: symptomatic mutation carriers

^11^C-DED binding in the temporal ROI showed a longitudinal significant negative association to plasma GFAP concentration in ADAD mutation carriers (Fig. 5A) while the ^18^F-FDG uptake in temporoparietal ROI and plasma GFAP concentration did not show a significant longitudinal association in ADAD mutation carriers (Fig. 5B). When evaluating the associations in all cortical ROIs, we observed that both ^11^C-DED binding in widespread cortical ROIs and ^18^F-FDG uptake in parietal ROIs showed significant negative longitudinal associations with plasma GFAP concentration in ADAD mutation carriers (Fig. 5C, D).

No longitudinal association was observed between plasma GFAP and the tracers’ binding/uptake in non-carriers.

## Discussion

**Fig. 6:**
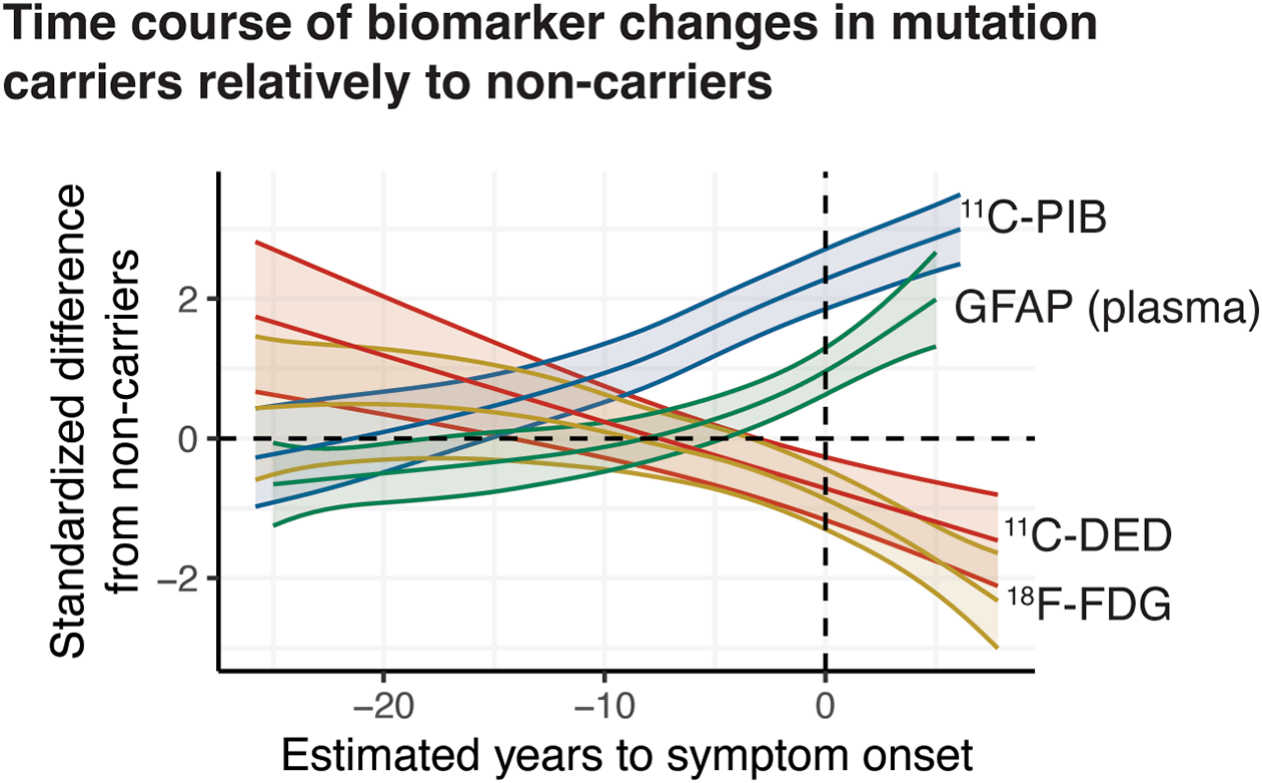
Longitudinal trajectories of biomarker changes based on generalized additive mixed-effects modelling in ADAD mutation carriers. Regression plots comparing the longitudinal nonlinear trajectories of the different biomarkers in ADAD mutation carriers across the estimated time to expected symptom onset; the biomarker values are presented as standardised difference from non-carriers (NC) and the shaded areas depict the 95% confidence interval for each regression curve. The ^11^C-DED, ^11^C-PIB and ^18^F-FDG binding/uptake were quantified in the composite temporal, global cortical and temporoparietal ROIs, respectively. pMC: presymptomatic mutation carriers; sMC: symptomatic mutation carriers

In the present study, we observed divergent trajectories of brain reactive astrogliosis, as imaged with ^11^C-DED, and plasma GFAP concentration, respectively, in carriers of ADAD mutations (Fig. 6). ^11^C-DED binding appears to be high in the very early presymptomatic stage of the disease and it later declines below the level of non-carrier controls. In the same carriers of ADAD mutations, the increase in plasma GFAP levels was first detectable at the time point that ^11^C-DED binding declines below the non-carrier levels. The time points when the level of the individual biomarkers in carriers of ADAD mutations show substantial differences from the non-carriers was similar to those of previous reports in studies that evaluated each biomarker separately [10, 19, 20]. Notwithstanding, the fewer follow-up points for modelling the PET tracer binding than the plasma GFAP trajectories, the findings suggest that reactive brain astrogliosis measured with ^11^C-DED binding and plasma GFAP concentration reflect distinct aspects of the Alzheimer’s disease pathology. Given the wide heterogeneity of astrocyte reactivity [34], however, one cannot exclude that the two biomarkers reflect different but associated states of astrocytes, especially given the strong longitudinal associations between the biomarkers. The results of this research in both participants with sporadic Alzheimer’s disease and carriers of ADAD mutations support in this case our two-wave hypothesis of astrogliosis in Alzheimer’s disease and that changes in ^11^C-DED binding reflect an early reactive astrogliosis wave [2, 35], which shows functional changes, such as changes in mitochondrial function, in the form of MAO-B overexpression [36], while plasma GFAP might reflect a later state of astrocytes, which shows overexpression and/or release of GFAP from their cytoskeleton [37]. A dynamic transition between the astrocyte activation states through cell remodelling could explain the observed association [4, 5]. The observed negative correlation between glucose metabolism and plasma GFAP concentration in this study together with the temporal differences, where changes in ^11^C-DED binding appear before changes in plasma GFAP and glucose metabolism, and earlier studies indicating that levels of GFAP might be associated with the degree of neuronal injury [5, 38, 39], give support to the hypothesis of a temporally later detection of GFAP released from astrocytes compared to detection of MAO-B overexpressing astrocytes. We have previously reported similar findings in an Alzheimer’s disease mouse model, where ^11^C-DED and GFAP follow divergent trajectories with a later detection of GFAP expression relatively to the detection of high ^11^C-DED binding [13]. Nevertheless, all above-mentioned theories remain to be proven, especially because the relationship between astrocyte GFAP levels and concentration of the protein in plasma is not known. Isoforms of GFAP are also produced in non-brain tissues [37, 40] and, furthermore, it is still unknown what proportion of brain GFAP crosses the blood-brain barrier, and whether other comorbidities contribute to the plasma protein concentrations, as shown recently with plasma phosphorylated tau measures [15]. Considerably more translational studies are required in order to shed light on the link between GFAP concentrations in the plasma and brain compartments.

The association between ^11^C-DED and plasma GFAP appears disease-specific in this study, given the absence of an association in non-carriers, despite that both ^11^C-DED binding and plasma GFAP showed detectable levels in this group. Earlier in vitro or postmortem studies have shown that astrocytes expressing MAO-B and GFAP could have a complex relationship, since inconsistent findings have been reported in different conditions [13, 41–45]. These results stress once more our limited understanding of astrocyte heterogeneity because of the sparsely available biomarkers and suggest a context-specific activation of astrocytes which has earlier been hypothesized [3, 34].

The existing literature on plasma GFAP is rather limited but has so far unanimously suggested that plasma GFAP is an early marker of amyloid-β pathology, based predominantly on cross-sectional data of individuals with sporadic AD [17, 46, 47]. We observed a positive correlation between levels of amyloid-β and plasma GFAP in our sample, similar to what has been shown earlier [17], although a large scatter was observed in the aforementioned correlation. The latter scatter could be explained by (1) a complex non-linear relationship between markers as earlier described [48], with negative sign especially in very high amyloid-β levels (in our sample, cases with very high amyloid-β were excluded when testing for this association), and (2) by the temporal dissociation that we observed between the increases in amyloid-β and plasma GFAP levels in ADAD mutation carriers. More specifically, amyloid-β levels rise above the average level of the non-carriers approximately 20 years before expected symptom onset [10], while the increases of plasma GFAP concentrations were detectable later, approximately 10 years before expected symptom onset [19], at a similar time point to when changes in glucose metabolism were observed [10]. The antiparallel temporal trajectories between changes in glucose metabolism and plasma GFAP concentration in our sample were further strengthened by the observed longitudinal association between markers, especially when looking at glucose metabolism in parietal areas, one of the areas demonstrating the earliest changes in this biomarker [49]. These findings support those of earlier studies in the same sample which suggest that levels of plasma GFAP increase later than the build-up of amyloid-β pathology (measured with PET/CSF markers) in the disease timeline, although plasma GFAP concentration increases are detectable at the presymptomatic stage of the disease [10, 19, 50], as replicated in another ADAD cohort [20].

In our sample, we observed parallel trajectories of reactive astrogliosis as imaged with ^11^C-DED and changes in glucose metabolism in ADAD mutation carriers, although the changes in ^11^C-DED binding appear to precede those in ^18^F-FDG. In our previous report, we demonstrated a positive longitudinal association between these two markers in the same participants [51] Although the exact source of this association remains unclear, it aligns with experimental evidence suggesting that reactive astrogliosis could contribute to the ^18^F-FDG signal [52].

The generalizability of these results is subject to certain limitations. Firstly, when comparing the levels of plasma GFAP concentration in our non-carrier group with control groups from earlier publications [17], we observed relatively higher concentrations in the non-carriers, displaying clusters of high and low values. However, when assessing the values in our larger non-carriers group independently of the presence or absence of ^11^C-DED data, the values were found to be comparable to those reported in other studies [19, 20]. The elevated levels observed in this non-carrier subgroup may be attributed to an uncontrolled selection bias, such as potential comorbidities, combined with the small sample size. This highlights the need for caution when interpreting data from non-carriers as controls, as the inclusion criteria for this rare group may be more flexible compared to the strict criteria often applied to healthy controls in biomarker studies. Secondly, it is essential to acknowledge that the use of linear modeling to illustrate the longitudinal trajectories of the biomarkers represents a simplistic approach, mainly due to the limited sample size. This approach can only capture a single phase of neuropathological changes and may not fully model the waves of reactive astrogliosis, as suggested by earlier studies from multiple research groups [2]. In other words, with the use of these models, we were unable to fully capture both the early increase and later decline of MAO-B load. To comprehend the complete dynamics of these biomarkers, more complex modeling techniques would be necessary, which would require larger sample sizes and longer follow-up periods. Thirdly, the existing evidence with ^11^C-DED supports the presence of early reactive astrogliosis in the trajectory of Alzheimer’s disease, with high binding observed at the early symptomatic stage (MCI) and low binding at the dementia stage [53]. Similar findings have been reported in humans using the ^18^F-DED [54] or a tracer targeting imidazoline I2 receptors [8], which are closely associated with MAO-B, and in Alzheimer’s disease mouse models with ^11^C-DED [13]. However, a study using the recently developed MAO-B tracer ^18^F-SMBT-1 showed somewhat contrasting results compared to those obtained with ^11^C-DED, as high ^18^F-SMBT-1 binding was detected even in patients with Alzheimer’s disease dementia [6]. These disparities in the findings can be attributed to differences in the design and methodologies employed in the studies. Firstly, the small sample size in all studies necessitates careful interpretation of the results for both tracers, particularly for ^18^F-SMBT-1, where the very limited number of individuals hinders more detailed comparisons within these often heterogeneous in terms of MAO-B load subgroups of patients with MCI + and Alzheimer’s disease [44]. Secondly, different methods were used to quantify the tracer binding. For ^11^C-DED, we utilized kinetic modeling with cerebellar grey matter as the reference region, which shows relatively sparse MAO-B concentrations [55]. On the other hand, for ^18^F-SMBT-1 static quantification was used with white matter as the reference region for maximizing the effect size between controls and patients with Alzheimer’s disease a posteriori, even though white matter has reported MAO-B loads [56, 57]. These factors limit the direct comparability of the studies using ^11^C-DED and ^18^F-SMBT-1, and therefore underscore the need to validate the methodologies used for quantifying MAO-B binding using arterial or image-derived input functions as standard of reference. All in all, we emphasize the requirement for studies with expanded sample sizes and prolonged follow-up intervals to provide a more comprehensive characterization of the trajectory of MAO-B associated astrocytes.

This study suggests that plasma GFAP concentration and PET ^11^C-DED brain binding follow divergent trajectories in ADAD mutation carriers. Regional ^11^C-DED binding concentrations were high decades before symptom onset and declined over time, while plasma GFAP levels rose above the non-carriers level closer to symptom onset but still in the presymtomatic phase. These results, together with the tight negative association between plasma GFAP concentration and ^11^C-DED PET, suggest that the two biomarkers may reflect distinct states/subtypes of reactive astrocytes that are strongly associated over time in both sporadic Alzheimer’s disease and carriers of ADAD mutations. Furthermore, our ADAD data and the longitudinal association between glucose metabolism and plasma GFAP changes suggest that plasma GFAP changes could be a later marker in the Alzheimer’s disease trajectory than earlier thought and could even reflect an Alzheimer’s disease-related degeneration of astrocytes. Future studies with the addition of more time points for each biomarker could better model the exact longitudinal trajectories of, and the association between each biomarker in the Alzheimer’s disease continuum. At the same time, more research is needed to validate plasma GFAP concentration as a robust marker of reactive astrogliosis.

### Electronic supplementary material

Below is the link to the electronic supplementary material.

Supplementary Material 1

## Data Availability

The largest number of participants in the present study are belonging to families with Autosomal Dominant Alzheimer Disease mutations, with most mutation carriers being at the presymptomatic stage of the disease. For protecting the confidentiality of the mutation status of the participants, the datasets are not available to the public since anonymization does not allow to always preserve confidentiality given the relatively small sample size. Anonymized data will be shared by request from a qualified academic investigator for replication of procedures and results presented in the article and if data transfer is in agreement with EU legislation on the general data protection regulation and decisions by the Ethical Review Board of Sweden. Sharing of data should be regulated in a material transfer agreement and or data processing agreement as appropriate.
