# Supplementary material for: Tracking reactive astrogliosis in autosomal dominant and sporadic Alzheimer’s disease with multi-modal PET and plasma GFAP
Source: Mol Neurodegener. 2023 Sep 12;18:60. doi: 10.1186/s13024-023-00647-y (PMC10496408; doi:10.1186/s13024-023-00647-y)
Supplement: Supplementary file 1 — Supplementary Material 1 [file 13024_2023_647_MOESM1_ESM.docx]

Supplementary Information

**Tracking astrocytes in autosomal dominant Alzheimer’s disease with plasma GFAP and multi-modal PET**

**Definition of amyloid-β status for patients with MCI or Alzheimer’s disease:**

A threshold of 1.41 SUVR with reference to the cerebellar grey matter was applied to the ^11^C-PIB PET images for defining amyloid-β positivity, based on previous work from our group ^1,2^.


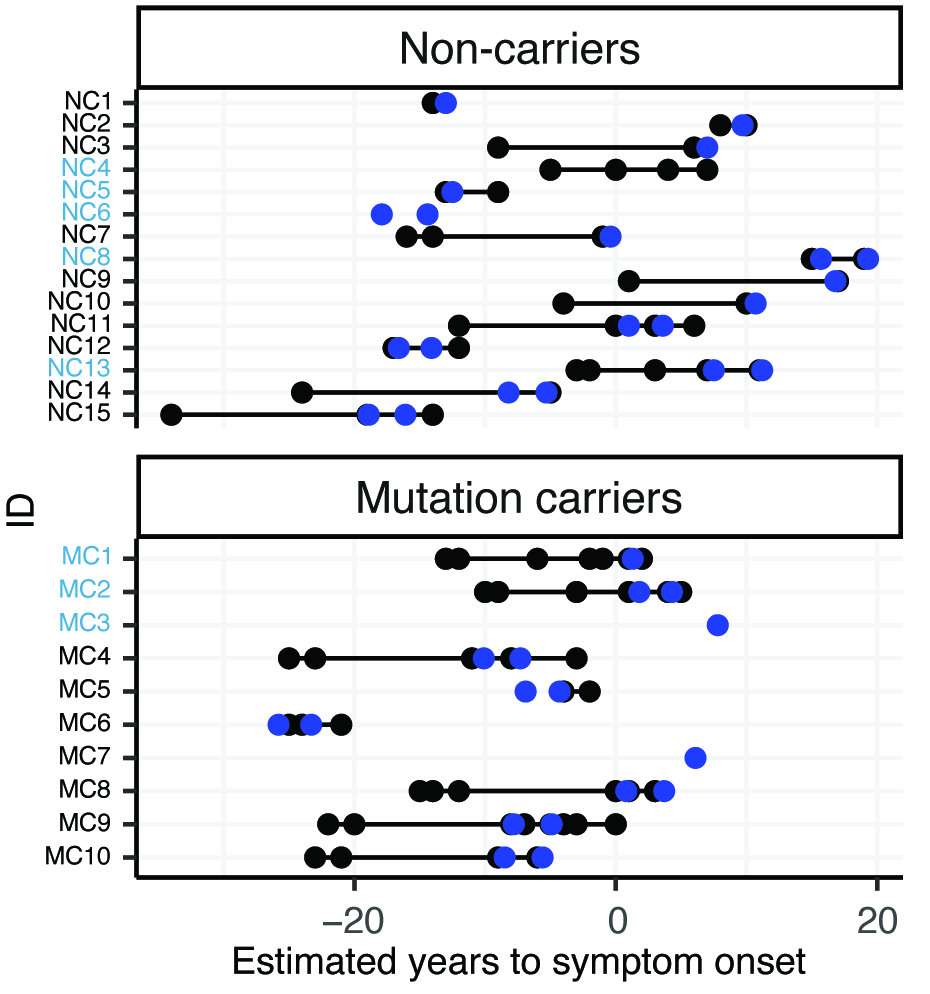


Supplementary Figure 1. Spaghetti plot illustrating the time points of plasma sampling (black) and PET acquisitions (blue) for every individual from the ADAD sample. The IDs of the members of families carrying APParc mutation are marked in blue. The carriers of the APParc gene were excluded for the analyses pertaining to ^11^C-PIB binding, due to the known mutation-specific relative sparsity of fibrillar amyloid-β that cause exceptionally low ^11^C-PIB binding levels.

**Patients with MCI and a negative amyloid-β PET scan:**

Four patients that qualified for a diagnosis of MCI had a negative amyloid-β PET scan. All of them were offered follow-up appointments at the Cognitive Clinic at Karolinska University Hospital. One individual did not attend the follow-up appointment. Two individuals remained cognitively stable over a follow-up period of 5-6 years (MMSE 29 -> 29 or MMSE 26 -> 25, respectively), and one individual developed a non-AD dementia syndrome over a follow-up period of 9 years.

**Investigations per individual:**

In Supplementary Figure 1, the reader can appreciate the time points of evaluations with plasma sampling and PET acquisitions in every individual patient from the ADAD sample. For the sporadic Alzheimer’s disease sample, only cross-sectional measures are presented in the manuscript for both plasma GFAP levels and PET quantification and are therefore excluded from this plot.

**Intraindividual variability analysis**:

The longitudinal trajectories of plasma GFAP for all participants were plotted across time after centring all longitudinal values to the baseline values for each participant (Supplementary Figure 2). We can observe that mutation carriers, except for one individual (longitudinal trajectory marked with *), demonstrated increases in their GFAP values, while no clear trend was observed in the non-carriers. The longitudinal trajectory of this individual (*) was deviating substantially from those of the other individuals, with a very steep decline between first and second time points and a moderate increase between second and third time points (GFAP = 257 pg/mL at baseline, 113 pg/mL four years from baseline and 132 pg/mL six years from baseline). This was also clear when applying linear modelling to the plasma values for each participant separately across the years to estimated symptom onset–all individuals with three or more GFAP time points were included and no testing for statistical significance was performed because of the low number of time points per individual. When comparing the resulting slopes of all individuals, we observed a very steep negative slope for this individual (marked with *) (slope=-0.30), which was the only outlier relative to the slopes of the other participants, which were positive (median group slope=0.10, interquartile range=0.02:0.13). The latter raises the possibility that the baseline GFAP value for this individual (marked with *; GFAP = 257 pg/mL), which drives the negative slope, could be related to a variability in the biomarker values that could not be explained based on our data. Although the inclusion or exclusion of the baseline point for this individual did not affect the linear mixed-effect model results, it was evident that the same baseline value was least explained by the models (highest residual values). Therefore, we selected to exclude it from the analyses presented in the current study as probably implausible.


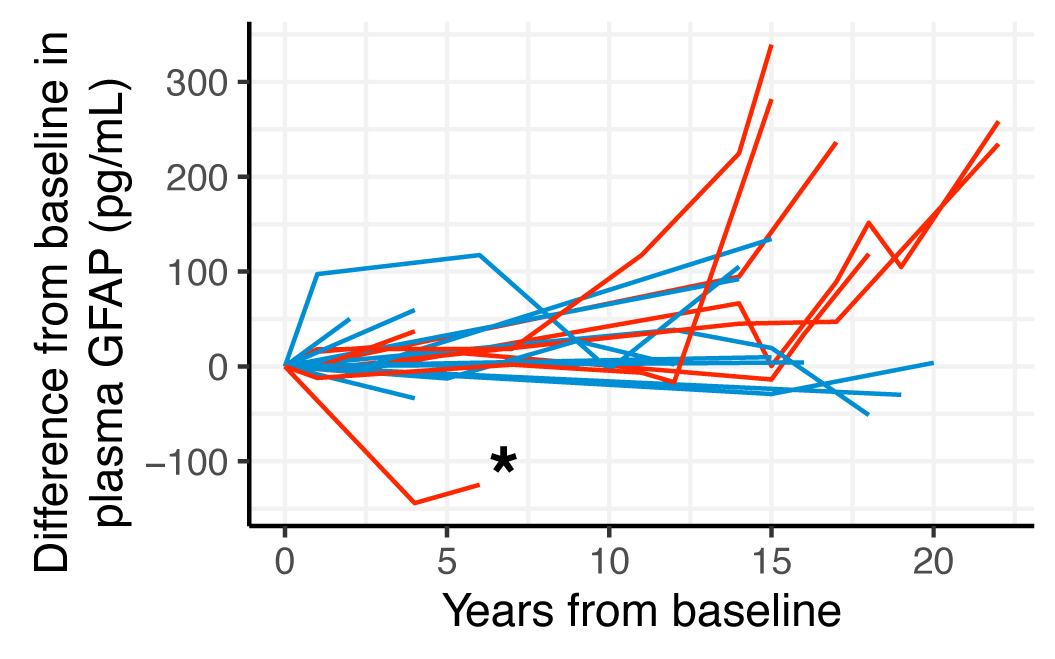


Supplementary Figure 2. Spaghetti plot illustrating the longitudinal changes in plasma GFAP in mutation carriers (red) and non-carriers (blue) across year from baseline.

Supplementary Figure 3. Scatterplots illustrating the association between ^11^C-DED binding as quantified by Patlak reference slopes and SUVR across all participants. The cerebellar grey matter was used as a reference region for both quantification methods.


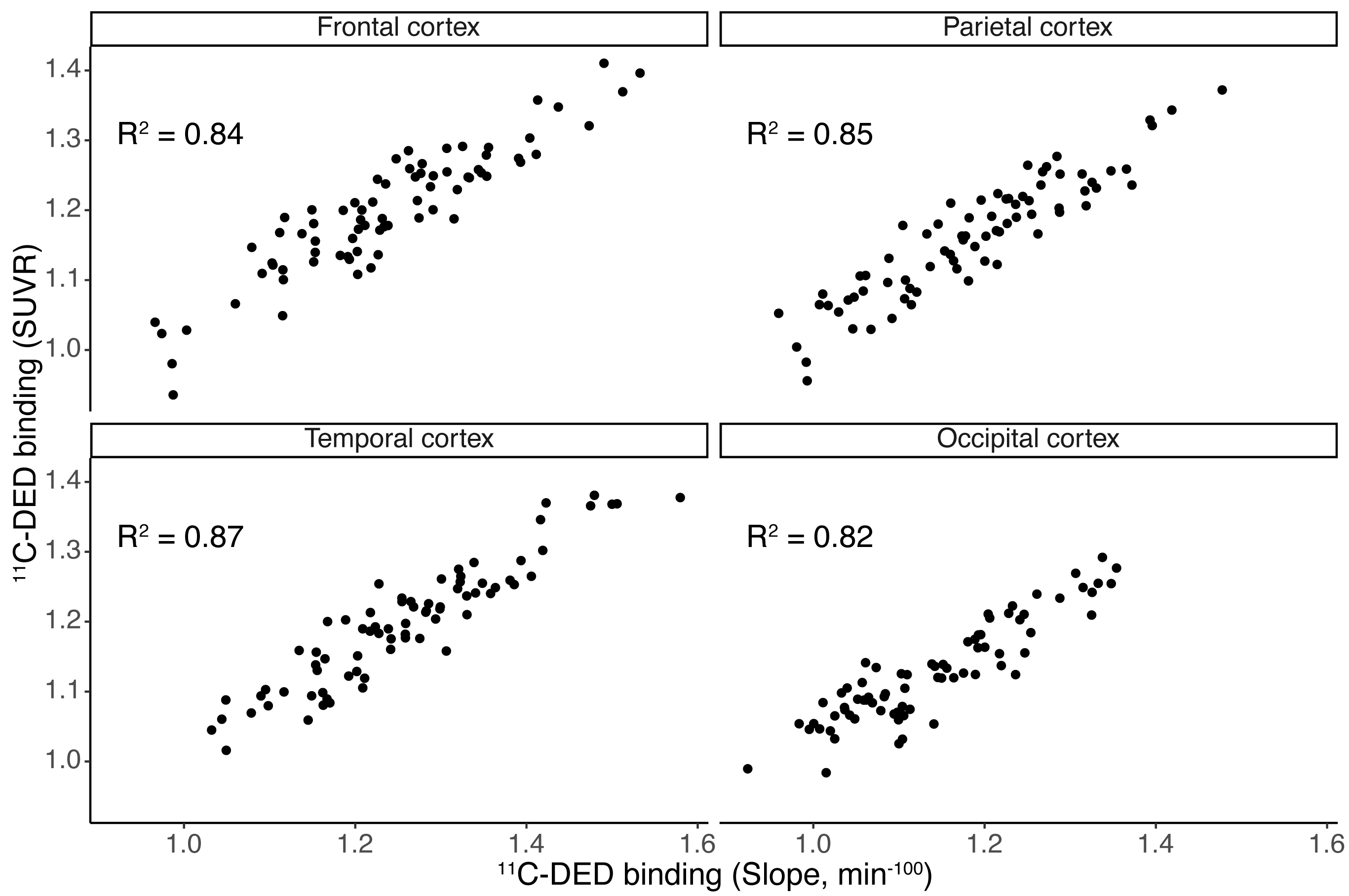


**^11^C-DED binding (Patlak slopes vs SUVR):**

We observed a strong correlation between Patlak slopes and SUVRs. Our interpretation suggests that the use of SUVRs may yield a reasonably accurate estimation of tracer binding. However, further investigation is necessary to fully characterize the comparability between static and dynamic quantification.

**Partial correlations:**

Similar results were obtained for the cross-sectional correlations between ^11^C-DED binding and plasma GFAP concentration, before and after applying correction for age and gender (partial correlations). After correction, the areas with significant correlations were slightly more restricted to fewer brain areas in the mutation carriers group, because of the lower degrees of freedom.

Supplementary Figure 4. Surface maps illustrating the areas with cross-sectional correlations between ^11^C-DED PET binding and plasma GFAP levels, before and after applying correction for age and gender, in non-carriers, carriers of ADAD mutations and patients with sporadic Alzheimer’s disease. Cortical ROIs whose binding show a significant association with plasma GFAP, are depicted in colour. MCI+: MCI with a positive amyloid-β PET scan; AD: Alzheimer’s disease dementia.


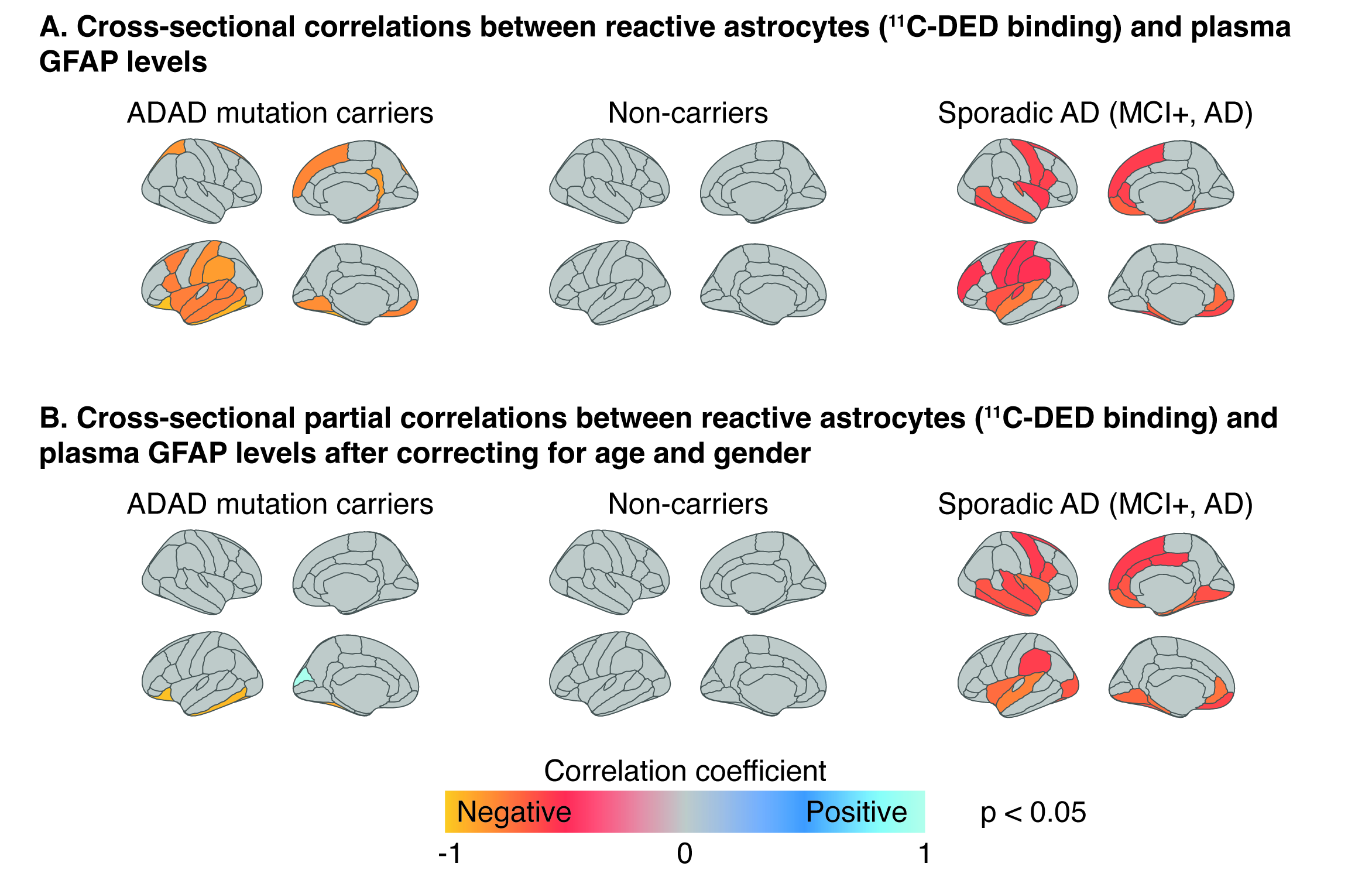


**References**

1. Rodriguez-Vieitez E, Saint-Aubert L, Carter SF, et al. Diverging longitudinal changes in astrocytosis and amyloid PET in autosomal dominant Alzheimer’s disease. *Brain*. 2016;139(3):922-936. doi:10.1093/brain/awv404

2. Nordberg A, Carter SF, Rinne J, et al. A European multicentre PET study of fibrillar amyloid in Alzheimer’s disease. *Eur J Nucl Med Mol Imaging*. 2013;40(1):104-114. doi:10.1007/s00259-012-2237-2
